# Ultrasonic Treatment Increases Extraction Rate of Common Bean (*Phaseolus vulgaris* L.) Antioxidants

**DOI:** 10.3390/antiox8040083

**Published:** 2019-04-01

**Authors:** Qiong-Qiong Yang, Ren-You Gan, Ying-Ying Ge, Dan Zhang, Harold Corke

**Affiliations:** Department of Food Science & Technology, School of Agriculture and Biology, Shanghai Jiao Tong University, Shanghai 200240, China; yangqiongqiong@sjtu.edu.cn (Q.-Q.Y.); gy1994@sjtu.edu.cn (Y.-Y.G.); zhang.dan@sjtu.edu.cn (D.Z.)

**Keywords:** sonication, phenolic compounds, antioxidant properties, extraction optimization, *Phaseolus vulgaris*

## Abstract

The feasibility of improving the extraction rate of common bean antioxidants by ultrasonic treatment was investigated. Scanning electron microscopy (SEM) and spectrum Fourier transform infrared spectrophotometer (FT-IR) analysis revealed that ultrasonic treatment substantially altered the cellular structure of common bean seed, resulting in increased surface area, eroded cell walls, and greater exposure of cellulose and hemicellulose. The highest antioxidant activity was obtained at optimal extraction conditions (68 min, 55% acetone, 36:1 liquid to solid ratio, 30 ℃, and 480 W) which were optimized by response surface methodology. In terms of the extraction rate of common bean antioxidants, ultrasound-assisted extraction (UAE) exhibits about seven-fold higher extraction efficiency than conventional solvent extraction (CSE). In addition, 10 phenolic compounds in the common bean extracts were detected and quantified by high performance liquid chromatography (HPLC), including protocatechuic acid, catechin, chlorogenic acid, epicatechin, ferulic acid, coumarin, rutin, myricetin, cinnamic acid, and genistein. In summary, ultrasonic treatment is an ideal candidate methodology for improving the extraction rate of common bean antioxidants.

## 1. Introduction

In recognition of the great importance of pulses for sustainable agriculture and human nutrition, Food and Agriculture Organization of the United Nations (FAO) officially proclaimed 2016 as “the International Year of Pulses”, aiming to draw attention to these important crops. Common beans (*Phaseolus vulgaris* L.) are the second most important bean species in the world after soybean [1]. Common bean seeds are mainly composed of carbohydrates, proteins, and dietary fiber, and are often also a good source of antioxidants [2]. Antioxidants of common beans show potential health benefits such as antitumor [3], anti-mutagenicity [4], antiproliferative [5], and antioxidant activities [6]. As a result of these health benefits and therapeutic effects, it is crucial to gain accurate information on the phenolic profile of antioxidants in common beans and the effect of extraction methods on phenolic level and composition [7].

The extraction of antioxidants from plants is most commonly carried out by conventional methods such as Soxhlet extraction and hydrodistillation based on different solvent extraction capabilities and the application of heat and/or shaking systems [8]. The most important drawbacks of conventional extraction methods are that they are time-consuming, consume high levels of solvents, and degrade thermally labile compounds [9]. As an alternative, ultrasound-assisted extraction (UAE) has been proposed as a replacement for such conventional extraction systems, since UAE can reduce extraction time, energy expenditure, solvent use, and instrument size, as well as allow room temperature extraction and offer ease of use [10,11]. In order to further expand the application of UAE, this study investigated the impact of ultrasonic treatment on the antioxidant properties of common beans.

Ultrasound-assisted extraction of antioxidants in common beans is influenced by several factors such as extraction solvent, extraction time, ultrasonic power, and solvent-to-solid ratio, which can be optimized to reduce process costs [12]. Response surface methodology (RSM) is a mathematical and statistical tool that can model various input factors to output responses, analyze the interactions between factors, and determine the optimal region of the factor levels [13]. Therefore, it is widely applied in the optimization and modeling of various bioactive compound extraction processes. They include the optimization of antioxidant phenolic compounds from brewer’s spent grain extraction conditions [14] and antioxidant compound from Maqui (*Aristotelia chilensis* [Mol] Stuntz) berries [15]. In addition, design of experiments (DOE) analysis is able to screen out the key factors that have a significant effect on the extraction process and provide a context for the RSM. Hence, in this study, we applied DOE/RSM to optimize the ultrasound-assisted extraction process of common bean antioxidants. We also made a comparison of extraction methods on antioxidant activity and phenolic composition in common bean extract. In addition, we investigated the ultrasound effect on common bean extract structures using scanning electron microscopy (SEM) and Fourier transform infrared spectrophotometry (FT-IR). This study should offer a valuable reference for choosing extraction methods to maximize antioxidants in grain legumes.

## 2. Materials and Methods

### 2.1. Material Preparation

Dried speckled kidney bean, a type of common bean (size, 1.03 ± 0.10 cm; weight, 0.81 ± 0.06 g; moisture content, 7.19 ± 0.35%) was purchased from Wal-Mart Stores in Kunming, Yunnan, China, and was produced in 2017. Prior to extraction, the dried beans were ground to fine powder using a laboratory-scale dry grinder (JYS-M01, Joyoung Co., Ltd., Jinan, China). The powder was sifted through a 40-mesh sieve to obtain kidney bean powder of particle size less than 500 μm and was stored at 4 ℃ and used within 1 month.

### 2.2. Ultrasonic-Assisted Extraction (UAE)

The ultrasonic treatment of common beans was performed in a tunable ultrasonic bath. The sample (0.5 g) and certain volume of extraction solvent were added to a 50 mL tube. Ultrasonic power, extraction time, and extraction temperature were controlled by the equipment panel. Here, the extraction solvent consisted of water and acetone in different ratios, and the detailed ratios, extraction time, extraction temperature, and ultrasonic power were based on experiment design charts. After extraction, the sample was centrifuged (10 min, 3000× *g*, 22 ± 1 ℃) and the supernatant was collected and stored at 4 ℃, and analyzed within 24 h.

### 2.3. Conventional Solvent Extraction (CSE)

In order to compare with UAE, CSE was carried out under the optimal extraction conditions obtained by response surface methodology. Samples (0.5 g) were extracted at 30 ℃ for 68 min, using 18 mL 50% acetone as solvents in an electric hot water bath (DK-S26, Jinghong Co., Shanghai, China). After extraction, the sample was centrifuged (10 min, 3000× *g*, 22 ± 1℃) and the supernatant was collected and stored at 4 ℃.

### 2.4. Design of Experiments (DOE)

A two-level factorial (default generators) design was applied to build the distribution matrix, which was generated by Minitab 17 software (Pennsylvania State University, Philadelphia, PA, USA). This design comprehensively examined the effects of ultrasonic time, extraction temperature, ultrasonic power, liquid-to-solid ratio, and acetone concentration on the common bean antioxidants and screened the key factors that have significant effects on the extraction process. The low and high levels were assigned as “−1” and “+1”, respectively, (Table 1) and the experiment matrix was shown in Table 2. All experiments were carried out in triplicate according to the experimental design, and the average antioxidant activities were considered as the responses, Y_1_ (ferric ion reducing antioxidant power (FRAP), μmol Fe (II)/g dry weight (DW)) and Y_2_ (2,2−diphenyl−1−picrylhydrazyl free radical scavenging activity (DPPH), μmol Trolox/g DW), respectively. The Pareto Chart of Standardized Effects was used to illustrate the main effect or interactive effect, which is considered to be statistically significant when its magnitude is larger than the other contrast column effects [16].

### 2.5. Response Surface Methodology (RSM)

Based on the DOE results, the extraction time (X_1_, 40-60-80 min), acetone concentration (X_2_, 40-50-60%), and liquid to solid ratio (X_3_, 30-35-40 mL/g) were selected as the major influential independent variables, and their related coded levels of independent variables were presented in Table 3. A 5^3^ central composite design was used to evaluate the effects of the three independent variables on the extraction efficiency of antioxidants, which were reflected by two dependent responses Y_1_ and Y_2_.

Design Expert 8.0.6 Trial software (Sta-Ease, Minneapolis, MN, USA) was used for data regression analysis and estimation of regression equation coefficients. An empirical model was obtained by correlating the measured responses with independent variables using multiple regression analysis. The following equation was used to predict the second−order response function for the experiments.

(1) $$Y=\beta_{0}+\sum_{i=1}^{n}\beta_{i}X_{i}+\sum_{\begin{array}{l} i=1\\ j>1 \end{array}}^{n-1}\sum_{j=2}^{n}\beta_{\mathrm{ij}}X_{i}X_{j}+\sum_{i=1}^{n}\beta_{\mathrm{ii}}X_{i}^{2}+\varepsilon$$

where Y is the measured responses, X_i_ and X_j_ are independent variables, and ε depicts error. β_0_, β_i_, β_ii_, and β_ij_ are the intercept term, coefficients, quadratic coefficients, and coefficients of interaction effects, respectively.

### 2.6. Antioxidant Activity

The antioxidant activities of common bean extracts were evaluated by DPPH free radical scavenging activity (DPPH) assay and ferric-reducing antioxidant power (FRAP) assay according to our previously published work [17], and the results were expressed as micromole Trolox equivalent per gram of dry weight samples (μmol TE/g DW) and μmol Fe (II)/g DW.

### 2.7. High Performance Liquid Chromatography (HPLC) Analysis

The supernatant obtained under optimal extraction conditions was collected and lyophilized and then dissolved in methanol for HPLC analysis. Major phenolic compounds in the common bean extracts were identified and quantified using Shimadzu LC-20AR HPLC system (Shimadzu, Kyoto, Japan) equipped with a Shim-pack GIS C18 reversed phase column (4.6 × 250 mm, 5 μm, Shimadzu, Kyoto, Japan), a binary pump, and a diode array detector (SPD-M20A, Shimadzu, Kyoto, Japan). The mobile phase consists of solvent A (0.1% (*v/v*) formic acid in water) and solvent B (0.1% (*v/v*) formic acid in methanol). The analysis was performed by a gradient elution program: 0 min, 5% B (*v/v*); 3 min, 10% B (*v/v*); 13 min, 28% B (*v/v*); 23 min, 45% B (*v/v*); 38 min, 70% B (*v/v*); 43 min, 90% B (*v/v*); 48–63 min, 95% B (*v/v*). The sample injection volume was 20 μL, and the flow rate was set at 1.0 mL/min. Phenolic compounds were identified by comparison with the retention time and UV spectra of standards, and their contents were calculated based on the peak area under the maximum absorbance and calibration curve of the corresponding standard at 10–100 μg/mL. The results were expressed as mg/100g DW.

### 2.8. Characterization of Raw and Treated Common Beans

Prior to characterization, the treated common bean slurries were freeze-dried to maintain their original structures as much as possible. Afterward, the lyophilized samples were preserved in a desiccator at ambient temperature. An FEI Sirion 200 field-emission scanning electron microscope (FEI Co., Hillsboro, Oregon, USA) was used to observe the surface structures of raw and treated samples. A spectrum Fourier transform infrared spectrophotometer (FT-IR, Perkin Elmer Inc., Beaconsfield, Buckinghamshire, England) was applied to analyze the infrared signatures of the raw and treated common beans.

### 2.9. Statistical Analysis

All the measurements were performed in triplicate, and the results were expressed as mean standard deviation (SD), with *p* value less than 0.05 defined as statistical significance. The statistical software Design Expert 8.06 (Sta-Ease, Minneapolis, MN, USA), IBM SPSS Statistics 19 (IBM, New York, NY, USA), Minitab 17 software (Pennsylvania State University, Philadelphia, PA, USA), and Origin 8.5 (OriginLab, Hampton, MA, USA) were used for statistical analysis.

## 3. Results

### 3.1. Selection of Statistically Significant Effects Using 2^k^ DOE Analysis

**Figure 1:**
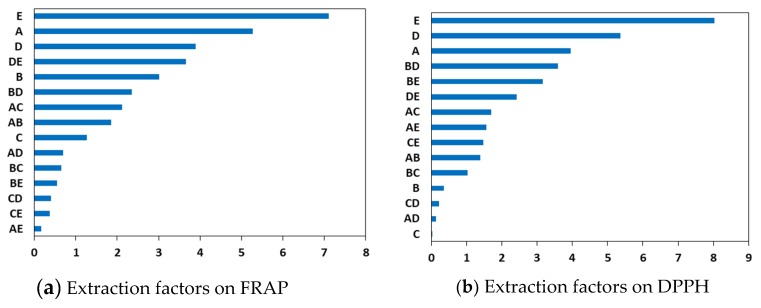
The main effects of ingredients on specified responses: (**a**) extraction factor effect on ferric−reducing antioxidant power (FRAP); (**b**) extraction factor effect on 2,2−diphenyl−1−picrylhydrazyl free radical scavenging activity (DPPH); A, extraction time; B, extraction temperature; C, ultrasonic power; D, liquid-to-solid ratio; E, acetone concentration.

DOE technique offers a structured approach for generating a significance test of factor levels and providing a prediction model for response. Here, a two-level factorial (default generators) design was used to build the distribution matrix and the results are shown in Table 2, which were further used to generate the Pareto Chart of Standardized Effects (Figure 1a,b). If the extraction factor increases from level “−1” to “+1”, the response also increases to the value of the corresponding effect, meaning that the extraction factor shows the main effect. Figure 1 shows that the top three extraction factors, including extraction time, liquid-to-solid ratio, and acetone concentration, were statistically significant for the two responses and should be studied further. These results can be used for the optimization of the following extraction process by RSM.

### 3.2. Fitting the Model

**Figure 2:**
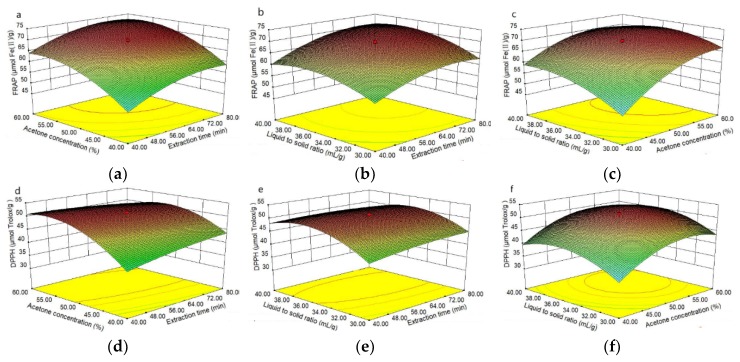
3D response surface curve and corresponding contour plot showing the effects of independent variables of ultrasonic treatment on DPPH and FRAP. (**a**) Mutual effects of acetone concentration and extraction time on FRAP; (**b**) mutual effects of liquid-to-solid ratio and extraction time on FRAP; (**c**) mutual effects of acetone concentration and liquid-to-solid ratio on FRAP; (**d**) mutual effects of acetone concentration and extraction time on DPPH; (**e**) mutual effects of liquid-to-solid ratio and extraction time on DPPH; (**f**) mutual effects of acetone concentration and liquid-to-solid ratio on DPPH.

Experimental modeling results for antioxidant activity (Table 4) show that FRAP and DPPH in common bean extracts varied from 41.1 to 70.1 μmol Fe(Ⅱ)/g DW and 30.3 to 51.7 μmol Trolox/g DW, respectively. The ANOVA results (Table 5) show that the model was highly significant (*p* < 0.0001) for FRAP and DPPH values. The lack of fit for both models was not significant (*p* > 0.05), which indicated that the established models fully explain the relationship between independent variables and responses. The *R*^2^ and adjusted *R*^2^ (Adj. *R*^2^) were close to 1, indicating a high degree of correlation between the predicted values and experimental values. Moreover, the predicted *R*^2^ (Pred *R*^2^) of each model was in reasonable agreement with the Adj. R^2^, indicating that the model predicts the response value very well. The Adeq Precision of each model is larger than 4, indicating that the model can be navigated in the design space. Furthermore, the coefficient of variance (CV) of each model was rather low (CV < 3%), which further supported the good adaptability of the model, thereby assuring better reproducibility. The resulting response surface 3D graph corresponding to each response is shown in Figure 2a–f.

### 3.3. Effects of Extraction Variables on Antioxidant Activity

ANOVA results showed significant linear (X_1_, X_2_, X_3_), quadratic (X_2_X_3_), and interactive ($X_{1}^{2}$, $X_{2}^{2}$, $X_{3}^{2}$) effects on FRAP (Table 5). Based on the regression coefficient (β) values (Equation (2)), X_2_ showed a major effect, followed by $X_{2}^{2}$, X_1_, $X_{3}^{2}$, $X_{1}^{2}$, X_3_, and X_2_X_3_. The linear effect of extraction time (X_1_), acetone concentration (X_2_), and liquid-to-solid ratio (X_3_) showed a highly significant (*p* < 0.0001) positive effect on FRAP; moreover, their quadratic terms ($X_{1}^{2}$, $X_{2}^{2}$, $X_{3}^{2}$) exhibited a highly significant (*p* < 0.0001) negative effect on FRAP. The interaction between the acetone concentration and liquid-to-solid ratio (X_2_X_3_) revealed a significant (0.0024) negative effect on FRAP (Table 5).

DPPH radical scavenging activity is largely determined by $X_{2}^{2}$, followed by $X_{3}^{2}$, X_2_, X_3_, X_1_X_2_, X_1_X_3_, and $X_{1}^{2}$ (Equation (3)). The linear effect of acetone concentration (X_2_) and liquid-to-solid ratio (X_3_) showed a highly significant (*p* < 0.0001) positive effect on DPPH, and their quadratic terms ($X_{2}^{2}$, $X_{3}^{2}$) exhibited a highly significant (*p* < 0.0001) negative effect on DPPH; moreover, $X_{1}^{2}$ also had a significant (*p* = 0.0210) negative effect on DPPH. The interaction between the extraction time and acetone concentration (X_1_X_2_) had a significant (*p* = 0.0004) negative effect on DPPH. The interaction between the extraction time and liquid-to-solid ratio (X_1_X_3_) revealed a significant (*p* = 0.0421) positive effect on DPPH (Table 5).

The models for antioxidant activities (FRAP and DPPH) were estimated by RSM, taking into account only the significant terms, which were shown in Equations (2) and (3):

(2) $$Y_{1}=69.42+4.56X_{1}+7.19X_{2}+3.29X_{3}-2.33X_{2}X_{3}-3.50X_{1}^{2}-4.97X_{2}^{2}-3.89X_{3}^{2}$$

(3) $$Y_{2}=51.15+4.12X_{2}+2.13X_{3}-2.04X_{1}X_{2}-0.91X_{1}X_{3}-0.80X_{1}^{2}-5.08X_{2}^{2}-4.74X_{3}^{2}$$

The non-significant values for lack of fit showed that the models were qualified with good prediction. In addition, the predicted *R*^2^ value (*R*^2^ = 0.9855 and 0.9875, respectively) fairly matched the adjusted *R*^2^ value (0.9725 and 0.9763, respectively) (Table 5).

In brief, acetone concentration (X_2_) and liquid-to-solid ratio (X_3_) revealed a positive effect for both responses. The interactive effects exhibited quite different patterns for the two responses (Y_1_ and Y_2_). Similar results were also obtained by others [18]. This may be attributed to the fact that the mechanism of the free radical reaction is different. Nevertheless, our results indicated that extraction time significantly influenced FRAP values, but had no significant effect on DPPH values.

### 3.4. Verification of the Predicted Optimal Extraction Conditions

In order to verify the reliability of the models, a single experiment was carried out under the modified optimal conditions: 68 min, 55% acetone, 36:1 liquid-to-solid ratio, 30 ℃, and 480 W. The experimental values for FRAP and DPPH were 75.2 μmol Fe(Ⅱ)/g DW and 50.9 μmol Trolox/g DW, which were well matched with the predicted values of 77.2 μmol Fe(Ⅱ)/g DW and 51.8 μmol Trolox/g DW with the coefficient of variance values of 2.68% and 1.84%, respectively.

### 3.5. Comparison of Extraction Methods on Antioxidant Activity and Phenolic Composition in Common Bean Extracts

Two extraction methods (UAE and CSE) were compared with regard to their extraction efficiency. These two extraction methods were performed under the same optimum extraction conditions obtained by central composite design (CCD) (68 min extraction time, 55% acetone, 36 mL/g, 30 °C).

In terms of extraction efficiency, UAE gave FRAP and DPPH values of 75.2 μmol Fe(Ⅱ)/g DW and 50.9 μmol Trolox/g DW, respectively, while CSE offered 9.87 μmol Fe(Ⅱ)/g DW (FRAP) and 7.76 μmol Trolox/g DW (DPPH), indicating that the UAE method yielded about eight-fold higher FRAP and about seven-fold higher DPPH than CSE method. Producing higher antioxidant activities through UAE can be attributed to the ultrasonic wave facilitating solvent penetration into the sample matrix and increasing the rates of mass transfer of antioxidants to the extraction solvent [19]. Similarly, in another study [20], UAE of antioxidants (phenolic compounds and anthocyanins) was compared with CSE, in terms of their respective efficiencies, concluding that the former provided a yield of about 2.5-fold higher total anthocyanin content and 3.2-fold higher total phenolic content than the latter. However, the antioxidant capacity of common beans using the whole seed was much lower than that in mung bean coats (178.28 μmol Trolox/g DW) obtained by ultrasound-assisted extraction in another study. As antioxidants mainly accumulate in seed coats, it is reasonable that it shows higher antioxidant capability. In addition, a similar conclusion was also obtained that UAE had higher extraction efficiency than CSE [21].

**Figure 3:**
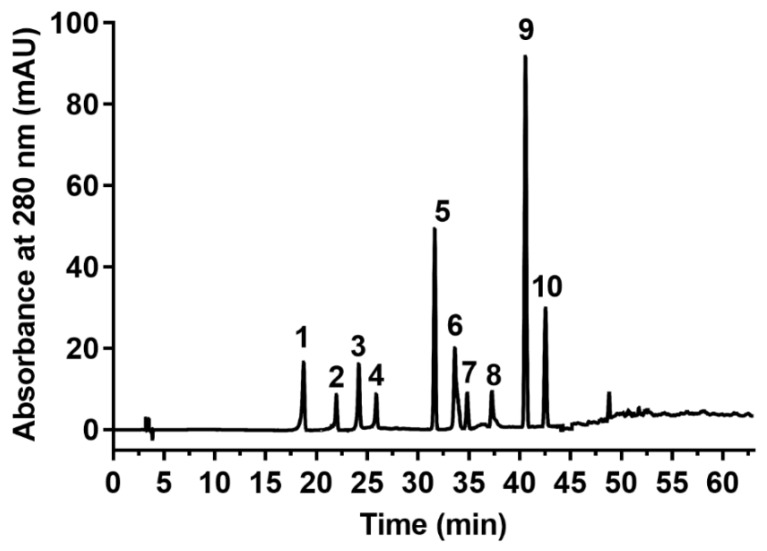
The chromatogram for the standards obtained by gradient elution on reversed phase C-18 column and Photo-Diode Array detector at 280 nm. Peak 1, protocatechuic acid; peak 2, catechin; peak 3, chlorogenic acid; peak 4, epicatechin; peak 5, ferulic acid; peak 6, coumarin; peak 7, rutin; peak 8, myricetin; peak 9, cinnamic acid; peak 10, genistein. mAU: miliabsorbance unit.

Ten polyphenolic antioxidant compounds in the extracts, including protocatechuic acid, catechin, chlorogenic acid, epicatechin, ferulic acid, coumarin, rutin, myricetin, cinnamic acid, and genistein, were detected and quantified by HPLC with standard curves. The chromatography of standards is shown in Figure 3. It is noteworthy that the contents of all phenolic compounds in the extracts obtained by UAE are higher than those obtained by CSE (Table 6), indicating that UAE has a higher extraction efficiency than CSE. A similar conclusion was also obtained by others [22]. Catechin and epicatechin recorded the two highest concentrations in UAE and CSE extracts, and the concentrations of catechin and epicatechin in UAE extracts were significantly higher than that in CSE extracts (Table 6). These levels were also higher than that in dark beans (*Phaseolus vulgaris* L.) [23]. In addition, compared to CSE, ultrasonic treatment also can significantly increase the chlorogenic acid, ferulic acid, cinnamic acid, genistein, and rutin levels, some of which were higher than that in other beans such as the concentration of ferulic acid (8.94 mg/100g DW) which obtained by UAE and was about ten-fold higher than that in dark beans (0.737 mg/100g DW) [24]. In addition, these phenolic compounds have also been detected in other common bean varieties and exhibit several health benefits [25]. Interestingly, the contents of chlorogenic acid (46.1 mg/100g DW) and ferulic acid (46.1 mg/100g DW) in common beans were significantly higher than those obtained by UAE in yellow soybean seeds (4.72–6.83 mg/100g DW and 1.42–5.82 mg/100g DW, respectively) [26]. Flavonoids such as catechin, epicatechin, rutin, myricetin, and genistein have shown significant biological activities including anti-bacterial, anti-tumor, and anti-inflammatory activities [25]. Phenolic acids such as protocatechuic acid, chlorogenic acid, ferulic acid, and cinnamic acid also exert several health benefits for oxidative stress-induced diseases via inhibiting lipid peroxidation, scavenging free radicals or chelating metal ions [27]. Therefore, the presence of phenolic compounds obtained under optimal UAE conditions makes it feasible for common beans to be a good source of functional food ingredients in industrial applications, especially in pharmaceuticals and nutraceuticals.

### 3.6. Effect of Ultrasonic Treatment on the Cellular Structure of Common Bean Seeds

**Figure 4:**
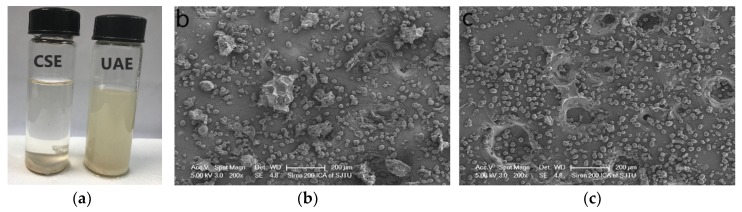
Picture and SEM images of the raw and treated common beans. (**a**) Picture of common beans in solvent before and after treatment; (**b**) raw common bean; (**c**) treated common bean.

Differences of the common bean slurries before and after ultrasonic treatment (Figure 4a) show enhanced dispersion. SEM showed that the surface morphology of the treated samples was significantly changed from that of the raw seeds (Figure 4b,c). In raw bean seeds, big aggregates were observed, which are starch granules surrounded by cotyledons and cell walls. However, few such aggregates were observed in treated beans, indicating that ultrasonication caused cell wall and cotyledon rupture. Ultrasonic treatment leads to a loose structure and erosion of the surface of common bean via cavitation that occurs near the surface [28]. This could result in more rapid and greater penetration of the solvent into the plant material. A similar conclusion was also obtained by others, who reported that ultrasound induces cavitation phenomena, has a mechanical mixing effect, and can cause structural changes in cellulose, such as increased surface area and decreased crystallinity [29,30].

**Figure 5:**
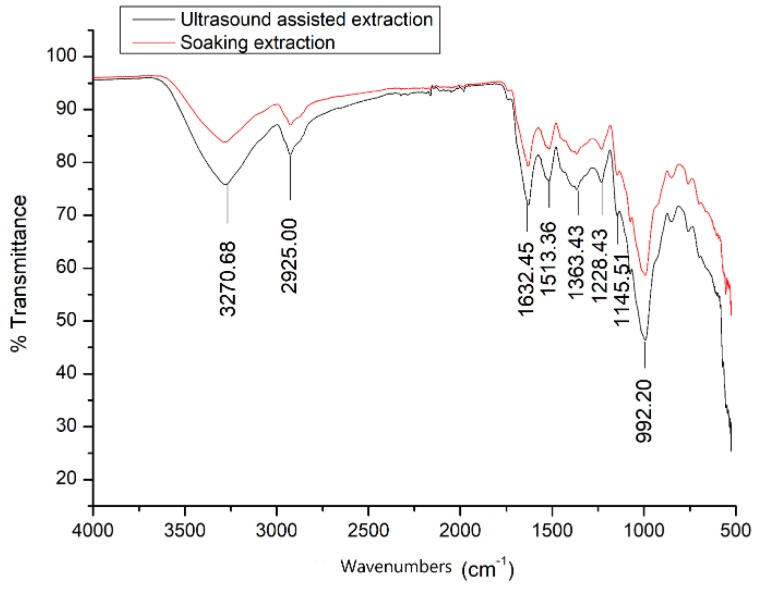
FT-IR spectra of the raw and treated common beans.

In addition to the physical effects of ultrasound, the sonochemical effect is another important factor in the change of the physicochemical properties of common beans. The sonochemical effects originate from a series of complex free radical reactions in water. Initially, ultrasonication cleaves the O–H bonds of H_2_O molecules and produces hydroxyl and hydrogen radicals, which then excite a chain reaction. The chain reaction produces more free radicals or oxidizing species such as hydrogen peroxide, oxygen, and ozone [31]. The oxidizing species can erode the solid structure of lignin, cellulose, and hemicellulose, leading to more exposure of granules, which can be confirmed by the treatment changes in the infrared signature of common beans (Figure 5). In treated common beans, the absorption peak at 3270 cm^−1^ was enhanced, and it represented the stretching vibration of O–H and hydrogen bonds in phenolic and aliphatic structures [32]. The enhanced absorption at 2925 cm^−1^ represented the stretch vibrations of the C–H bond in the methylene of cellulose [30,33]. The intensity ratio of the peaks at 1632 and 1513 cm^−1^ change, which may be related to the demethylation of the syringyl structure of lignin [34]. The enhanced peak at 1145 cm^−1^ is the C–H signal in planar deformation of glucosyl group in lignin [35]. These results indicate that ultrasonic treatment causes partial decomposition of the lignin network structure. In addition, ultrasonic treatment significantly increased the intensity of the peak at 992 cm^−1^, which is assigned to C–O stretching and is likely the C–O bond of cellulose and hemicellulose [30]. This indicates that ultrasonic treatment breaks the lignin to a certain extent and releases more cellulose and hemicellulose on the surface of common beans, which can be helpful for increasing the release of antioxidants.

## 4. Conclusions

In this study, ultrasound-assisted extraction of common bean antioxidants was optimized by RSM. The optimal extraction parameters were determined, and the predicted values were close to the experimental values. The optimal UAE conditions for common bean antioxidants included an extraction time of 68 min, extraction solvent concentration of 55%, liquid-to-solid ratio of 36 mL/g, extraction temperature of 30 ℃, and ultrasonic power of 480 W. Under these optimal conditions, the maximum FRAP and DPPH values of common beans were 75.2 μmol Fe(Ⅱ)/g DW and 50.9 μmol Trolox/g DW, respectively.

Compared to conventional extraction methods, ultrasonic treatment significantly increases the extraction rate of antioxidants, which were further analyzed using HPLC, and 10 phenolic compounds were detected, including protocatechuic acid, catechin, chlorogenic acid, epicatechin, ferulic acid, coumarin, rutin, myricetin, cinnamic acid, and genistein. In addition, the effects of ultrasound on common bean structures were analyzed using SEM and FT-IR, finding that ultrasonic treatment led to a loose structure and erosion of the surface of common beans, which could result in more rapid and greater penetration of the solvent into the plant material.

## Figures and Tables

**Table 1:** Experimental design factors and levels of design of experiments.

Levels	A: Ultrasonic Time (min)	B: Extraction Temperature (°C)	C: Ultrasonic Power (W)	D: Liquid to Solid Ratio (mL/g)	E: Acetone Concentration (%)
−1	40	30	400	30	40
1	80	50	560	40	60

**Table 2:** Experimental design and results of design of experiments.

No.	A (min)	B (℃)	C (W)	D (mL/g)	E (%)	FRAP (μmol Fe^2+^/g DW)	DPPH (μmol Trolox/g DW)
1	40	30	400	30	60	41.7 ± 0.26	39.7 ± 0.26
2	80	30	400	30	40	32.5 ± 0.34	27.9 ± 0.74
3	40	30	560	40	60	39.9 ± 0.95	45.2 ± 0.87
4	80	30	560	30	60	43.1 ± 1.00	40.4 ± 0.83
5	80	50	560	30	40	40.0 ± 0.52	36.7 ± 0.90
6	40	50	560	40	40	35.4 ± 0.78	32.6 ± 0.40
7	40	50	400	30	40	34.0 ± 0.41	31.0 ± 0.79
8	80	30	560	40	40	42.9 ± 1.00	39.0 ± 0.36
9	40	30	400	40	40	41.3 ± 0.36	37.4 ± 0.20
10	40	50	400	40	60	42.7 ± 0.85	37.0 ± 1.03
11	80	50	400	30	60	49.3 ± 1.18	40.0 ± 0.69
12	40	30	560	30	40	27.6 ± 0.26	21.5 ± 0.15
13	80	30	400	40	60	45.8 ± 0.94	45.3 ± 0.92
14	80	50	400	40	40	44.8 ± 0.79	39.5 ± 0.81
15	40	50	560	30	60	43.2 ± 0.86	39.0 ± 0.38
16	80	50	560	40	60	49.7 ± 0.95	43.3 ± 0.76

A, extraction time; B, extraction temperature; C, ultrasonic power; D, liquid to solid ratio; E, acetone concentration. FRAP: ferric ion reducing antioxidant power; DW: dry weight; DPPH: 2, 2−diphenyl−1−picrylhydrazyl free radical scavenging activity.

**Table 3:** Independent process variables with experimental ranges and levels of response surface methodology.

Independent Variable	Code				
	−1.68 (–α)	−1	0	+1	+1.68 (–α)
Extraction time (min)	26.36	40	60	80	93.64
Solvent concentration (%)	33.18	40	50	60	66.82
Liquid-to-solid ratio (mL/g)	26.59	30	35	40	43.41

**Table 4:** Experimental design with response of independent variables.

NO. ^a^	Independent Variables ^b^				Y_1_: FRAP (μmol Fe^2+^/g DW)			Y_2_: DPPH (μmol Trolox/g DW)	
	X_1_ (min)	X_2_ (%)	X_3_ (mL/g)		Experimental Data	RSM Predicted Results		Experimental Data ^c^	RSM Predicted Results
1	−1	−1	−1		41.1 ± 2.65 ^c^	39.9		30.5 ± 0.87	30.5
2	1	−1	−1		46.8 ± 2.55	48.7		36.6 ± 0.80	37.6
3	−1	1	−1		59.0 ± 2.55	59.7		42.3 ± 0.78	43.3
4	1	1	−1		68.6 ± 1.17	66.9		41.8 ± 1.11	42.2
5	−1	−1	1		48.2 ± 0.13	50.0		36.5 ± 0.61	37.1
6	1	−1	1		61.7 ± 1.82	61.0		40.5 ± 0.21	40.5
7	−1	1	1		62.3 ± 1.00	60.5		48.9 ± 0.29	48.9
8	1	1	1		68.6 ± 0.53	70.0		43.2 ± 0.88	44.2
9	−α	0	0		51.5 ± 0.48	51.9		48.4 ± 0.38	47.9
10	α	0	0		67.7 ± 2.65	67.2		50.8 ± 0.87	49.9
11	0	−α	0		44.3 ± 0.95	43.3		30.3 ± 0.49	29.9
12	0	α	0		66.6 ± 0.32	67.5		44.7 ± 0.62	43.7
13	0	0	−α		52.6 ± 2.65	52.9		35.1 ± 0.87	34.2
14	0	0	α		64.4 ± 2.42	64.0		41.9 ± 0.14	41.3
15	0	0	0		69.8 ± 0.36	69.5		51.5 ± 1.06	51.2
16	0	0	0		70.0 ± 1.06	69.5		51.5 ± 0.62	51.2
17	0	0	0		69.9 ± 1.21	69.5		51.7 ± 0.14	51.2
18	0	0	0		69.9 ± 0.12	69.5		51.5 ± 0.14	51.2
19	0	0	0		70.1 ± 0.64	69.5		51.5 ± 0.01	51.2
20	0	0	0		66.9 ± 0.24	69.5		49.0 ± 0.57	51.2

^a^ Experimental conditions according to central composite design (CCD); ^b^ X_1_: Extraction time, X_2_: Acetone concentration, X_3_: liquid-to-solid ratio; ^c^ Experimental values: mean ± S.D. (*n* = 3).

**Table 5:** Results of ANOVA about central composite design ^a^.

Variables	FRAP						DPPH				
	Sum of Squares ^b^	Df ^c^	Mean Square ^d^	F-Value ^e^	*p*-Value ^f^		Sum of Squares	Df	Mean Square	F−Value	*p*-Value
Model	1815.89	9	201.77	75.54	<0.0001		971.38	9	107.93	87.95	<0.0001
X_1_ (β_1_)	284.18	1	284.18	106.39	<0.0001		4.50	1	4.50	3.66	0.0847
X_2_ (β_2_)	706.37	1	706.37	264.45	<0.0001		231.41	1	231.41	188.56	<0.0001
X_3_ (β_3_)	148.27	1	148.27	55.51	<0.0001		61.99	1	61.99	50.51	<0.0001
X_1_X_2_ (β_1_β_2_)	1.27	1	1.27	0.48	0.5058		33.21	1	33.21	27.06	0.0004
X_1_X_3_ (β_1_β_3_)	2.45	1	2.45	0.92	0.3605		6.66	1	6.66	5.43	0.0421
X_2_X_3_ (β_2_β_3_)	43.38	1	43.38	16.24	0.0024		0.45	1	0.45	0.37	0.5578
$X_{1}^{2}$ (β_11_)	176.14	1	176.14	65.94	<0.0001		9.19	1	9.19	7.49	0.0210
$X_{2}^{2}$ (β_22_)	355.51	1	355.51	133.10	<0.0001		371.38	1	371.38	302.62	<0.0001
$X_{3}^{2}$ (β_33_)	217.90	1	217.90	81.58	<0.0001		324.35	1	324.35	264.29	<0.0001
Lack of fit	19.16	5	3.83	2.54	0.1650		6.80	5	1.36	1.24	0.4086
*R* ^2^	0.9855						0.9875				
Adj *R*^2^	0.9725						0.9763				
Pred *R*^2^	0.9021						0.9378				
Adeq Precision	26.043						27.167				
C.V. %	2.68%						2.52%				

^a^ The results were obtained with Design Expert 8.0.6 software; ^b^ Sum of the squared differences between the average values and the overall mean; ^c^ Degrees of freedom; ^d^ Sum of squares divided by degree of freedom; ^e^ Test for comparing term variance with residual variance; ^f^ Probability of the observed F-value, *p* < 0.05 defined as significant.

**Table 6:** Phenolic profile of common bean extracts under optimal extraction condition.

No.	Compound	Retention Time (min)	Concentration (mg/100g DW)	
			UAE ^a^	CSE ^b^
1	Protocatechuic acid	18.7	177 ± 5.09	170 ± 3.09
2	Catechin	22.0	317 ± 6.06	297 ± 6.13 *
3	Chlorogenic acid	24.2	46.1 ± 1.05	41.0 ± 1.23 *
4	Epicatechin	25.9	117 ± 3.12	101 ± 3.13 *
5	Ferulic acid	31.6	8.94 ± 0.13	8.09 ± 0.15 *
6	Coumarin	33.6	10.2 ± 0.47	10.1 ± 0.46
7	Rutin	34.8	19.7 ± 0.95	15.8 ± 0.89 *
8	Myricetin	37.3	32.3 ± 1.19	30.1 ± 1.25
9	Cinnamic acid	40.6	0.25 ± 0.22	0.21 ± 0.19 *
10	Genistein	42.5	4.74 ± 0.35	3.64 ± 0.36 *

* *p* < 0.05; ^a^ Ultrasound-assisted extraction; ^b^ Conventional solvent extraction.
